# The Critical Role of Supervision in Retaining Staff in Obstetric Services: A Three Country Study

**DOI:** 10.1371/journal.pone.0058415

**Published:** 2013-03-21

**Authors:** Eilish McAuliffe, Michael Daly, Francis Kamwendo, Honorati Masanja, Mohsin Sidat, Helen de Pinho

**Affiliations:** 1 Centre for Global Health & School of Medicine, University of Dublin, Trinity College, Dublin, Ireland; 2 School of Management, University of Stirling, Stirling, United Kingdom; 3 University of Malawi, College of Medicine, Centre for Reproductive Health, Blantyre, Malawi; 4 Ifakara Health Institute, Dar Es Salaam, Tanzania; 5 Department of Community Health, Faculty of Medicine, Eduardo Mondlane University, Maputo, Mozambique; 6 Averting Maternal Death and Disability Program, Heilbrunn Department of Population and Family Health, Mailman School of Public Health, Columbia University, New York, New York, United States of America; Tehran University of Medical Sciences, Iran (Islamic Republic of Iran)

## Abstract

Millennium Development Goal (MDG) 5 commits us to reducing maternal mortality rates by three quarters and MDG 4 commits us to reducing child mortality by two-thirds between 1990 and 2015. In order to reach these goals, greater access to basic emergency obstetric care (EmOC) as well as comprehensive EmOC which includes safe Caesarean section, is needed.. The limited capacity of health systems to meet demand for obstetric services has led several countries to utilize mid-level cadres as a substitute to more extensively trained and more internationally mobile healthcare workers. Although this does provide greater capacity for service delivery, concern about the performance and motivation of these workers is emerging. We propose that poor leadership characterized by inadequate and unstructured supervision underlies much of the dissatisfaction and turnover that has been shown to exist amongst these mid-level healthcare workers and indeed health workers more generally. To investigate this, we conducted a large-scale survey of 1,561 mid-level cadre healthcare workers (health workers trained for shorter periods to perform specific tasks e.g. clinical officers) delivering obstetric care in Malawi, Tanzania, and Mozambique. Participants indicated the primary supervision method used in their facility and we assessed their job satisfaction and intentions to leave their current workplace. In all three countries we found robust evidence indicating that a formal supervision process predicted high levels of job satisfaction and low intentions to leave. We find no evidence that facility level factors modify the link between supervisory methods and key outcomes. We interpret this evidence as strongly supporting the need to strengthen leadership and implement a framework and mechanism for systematic supportive supervision. This will promote better job satisfaction and improve the retention and performance of obstetric care workers, something which has the potential to improve maternal and neonatal outcomes in the countdown to 2015.

## Introduction

The provision of high quality health services depends upon the presence and performance of skilled personnel [1]. Yet 57 countries, 36 of which are in sub-Saharan Africa, face a critical shortage of health workers [2] and will struggle to meet the health Millennium Development Goals by 2015. This gap in services urgently needs to be addressed, in a manner that ensures more equitable distribution of health workers with context-specific skills [3]. Much of the literature is in agreement that the delivery of quality health services in resource poor countries requires teams of health professionals with varied skill sets appropriate to the particular work context [e.g. 4]. This is hampered by the challenge of retaining workers, although there is some evidence to suggest that the use of initiatives to improve motivation have been effective in helping retention [5]. The importance of context-specific motivators is highlighted in a study of public and private sector healthcare providers in two Indian states which highlights the need to locally assess conditions and manage incentives to ensure health workers are motivated in their work [6].There is also growing awareness of the importance of the psychosocial work environment for the delivery of high quality care [7]. Healthcare workers under stress are more likely to treat patients poorly, both medically and psychologically and are also more prone to make errors of judgment [7]. In the provision of emergency obstetric care the survival of mother and neonate is dependant on decisive rapid action in the presence of complications, such as obstructed labour. Recent evidence suggests that the rate of caesarian sections in low-income settings is at the lower end of the WHO recommended optimum range of 5–15% and that timely referral for caesarian sections could have a significant positive impact on maternal mortality rates [8]

Many countries are working to expand health services to meet population need, by utilizing mid-level providers as an essential component of healthcare teams [9], [10]. Mid-level providers undertake roles and tasks which are normally the responsibility of established internationally recognized cadres, such as doctors and nurses [9]. There is evidence that mid-level providers are effective in service delivery, particularly in the provision of emergency obstetric care [11]. However, maintaining the effectiveness of mid-level cadres is contingent on supportive working environments and the successful delegation of tasks [12]. Adequate supportive supervision underpins the task shifting process and is critical to quality in service delivery. Several studies of mid-level healthcare workers suggest that supervision is frequently absent and that even when present supervision may be solely corrective in nature (e.g. [10]).

Bradley and McAuliffe [13] report the supervision of these cadres in Malawi to be ad hoc and focused on the correction of staff errors, thus serving to demotivate rather than motivate health workers, with negative consequences for attrition. In contrast, supporting workers through regular scheduled supervision meetings is likely to assist workers in: gaining insights from appropriate feedback, realizing skill-gaps and potential training opportunities, understanding performance expectations, and setting professional development goals. Together these enhancements could improve job satisfaction and retention and improve the quality of service delivery. A systematic review by Cummings et al. [14] concluded that people focused leadership practices (including supportive supervision) contribute to improving work environments and the productivity and effectiveness of healthcare organizations.

In this study we identify the implications of different types of supervision for healthcare worker job satisfaction and intention to leave the workplace in Malawi, Tanzania, and Mozambique. Primarily, we expect negative outcomes when a supervision system is absent or is characterized principally by negative feedback. Our hypothesis is that when healthcare workers receive pre-arranged formal supervision at regular intervals this will be associated with elevated levels of job satisfaction [15] and diminished intentions to leave [16].

Our analyses use the Health Services Strengthening for Equity (HSSE) study data. The HSSE is a large-scale cross-sectional survey of healthcare providers that includes comprehensive measures of both facility and healthcare provider characteristics. It thus provides an ideal opportunity to identify if a relation between the supervision method employed and key outcomes is due to potentially confounding variables. In particular, in this study we utilize multilevel analysis to adjust for dependencies in data sampled from healthcare workers in the same facility and to identify the extent to which facility characteristics (e.g. geographic isolation, size, availability of resources) may influence the link between supervision and the outcomes of interest.

## Methods

This study is a cross-sectional descriptive study of healthcare facilities and healthcare providers in obstetric care in Malawi, Tanzania and Mozambique.

### Ethics Statement

The study was approved by the Institutional Review Board of Columbia University, New York; Global Health Ethics Committee Trinity College, Dublin; and the Institutional review boards of College of Medicine, Malawi, Eduardo Mondlane University, Mozambique and Ifakara Health Institute, Tanzania.

### Data Collection

Data were collected from healthcare providers and healthcare facilities between October and December 2008. Providers who indicated they had performed basic or emergency obstetric care tasks in the prior three months were eligible for participation. In Malawi, a near-national sample of facilities (N = 84) intended to provide EmOC services was identified and included central, district, rural and CHAM (faith-based organisations) –operated hospitals and a randomly sampled urban and recently upgraded health centres designated to provide EmOC. A few districts/facilities were excluded in Malawi due to their recent participation in another human resources study in which similar data had been collected from health workers.

In Tanzania, due to the size of the country, cluster sampling was employed. One region was randomly selected in each of the eight geographic zones and all districts within those eight regions were then included in the sampling frame. The primary hospital serving the district was identified for inclusion; either the government-run district hospital or voluntary agency-run (VA) designated district hospital (DDH). In some districts that also contain the regional headquarters, the regional hospital was included in the sample when there was no district hospital serving the community. One health centre (HC) was randomly selected in each district, thus there were two facilities from each district in the study (N = 90).

In Mozambique, a near national sample of general, district and rural hospitals was included to maximise the potential participation of the NPC cadre tecnico de cirurgia. In addition, two to three health centres (type 1 and type 2) providing maternity care, and therefore at least some basic EmOC functions, were randomly selected in each district for inclusion in the study (N = 138). Facilities were sampled from all rural regions outside Maputo City, as mid-level cadres such as surgical technicians are concentrated primarily in health facilities in rural regions with obstetricians and nurse midwives being concentrated in the Maputo City area.. Selected facilities were similar within and across the three countries and therefore the different selection approaches are unlikely to have influenced the results. Eligible providers were given detailed information about the study and its requirements and signed a consent form if they wished to participate. The actual response was limited by the numbers of eligible staff actually available in the facilities at the time the facility was visited and the data collector's efforts to ensure minimum disruption to health service delivery.

The facility survey was completed by the data collection team who compiled information on key facility metrics such as the number of beds in the facility and the availability of equipment and other resources. The team was assisted in this process by the facility and maternity in-charge and specific members of staff with expertise or access to records in the relevant area. The availability and functionality of equipment was confirmed through visual inspection. Data from the detailed facility level survey for Mozambique was not available at the time of writing and facility level analyses thus utilize the Malawi and the Tanzania data.

### Measures

#### Supervision method & adequacy of supervision

Participants indicated which of five methods of supervision best described the supervision experienced at their healthcare facility-“Formal supervision process with regular pre-arranged supervision meetings”, “Supervision is available if I request it from my line manager”, “Supervision consists of negative feedback when performance is poor”, “ I never receive any supervision or feedback on my performance”, or “other” form of supervision. These categorisations were derived from informal discussions with ministry and district/council level staff.

#### Job satisfaction

Job satisfaction was assessed using 5-items derived from a previously validated 7-item scale [17], [18]. Two items from the scale were dropped as they assessed satisfaction with supervision and were likely to inflate any estimates of the relationship between supervision methods and job satisfaction. The remaining items were summated (e.g. “In general, I am satisfied with this job”, “I am satisfied with my pay compared to similar jobs in other organizations”) and compiled scores on the augmented job satisfaction scale ranged from 5 (low job satisfaction) to 25 (high job satisfaction).

#### Intentions to leave

Three items were used to assess the likelihood that participants would leave their current position-“would consider working for another hospital/clinic”, “seriously thought about leaving this hospital/clinic”, and “actively seeking other employment”. On a 5-point Likert scale total scores ranged from 3 (low intention to leave) to 15 (high intention to leave).

#### Demographic and occupational characteristics and rationale for their inclusion

It is possible that certain demographic and occupational factors like age, gender, and cadre may impact on independent variables such as supervision methods and dependent variables like job satisfaction. Under this rationale we thus include age, gender, and cadre as covariates in all analyses.

#### Facility level variables and rationale for their inclusion

Even after adjustment for demographic and occupational characteristics it is possible that facility level factors may confound relationships between supervision methods and healthcare worker outcomes. In the HSSE study, comprehensive facility level information was collected from all facilities sampled in Malawi and Tanzania. Although the aim was to collect similar information in Mozambique some errors occurred in collection and coding that prevented the matching of individual and facility level data and thus it has been excluded from this analysis.

We include metrics of hospital size, geographic isolation and the availability of resources in all multilevel analyses conducted using data from Malawi and Tanzania. Hospital size was estimated from the number of beds recorded in the facility. Geographic isolation was indexed using the distance to the nearest referral hospital. Finally, the presence of ten key resources was recorded for each facility in order to gauge the adequacy of the facilities and resources available (e.g. availability of: electricity, clean water, staff room, meals for staff, staff toilet facilities, allowances for overtime work).

### Data analyses

The outcome variables, job satisfaction and intention to leave, were treated as continuous variables and predicted using linear multilevel modeling. Due to the hierarchical structure of the data with healthcare workers nested within facilities multilevel random coefficient modeling was deemed to be the most appropriate technique to answer most of the study questions [19]. This analytic method allows for uneven number of assessments per facility and estimates random variation in both the sampling of facilities and the sampling of workers within those facilities.

The analytic strategy for the multilevel analyses was as follows: firstly we estimate two separate random intercepts models using supervision methods to predict intentions to leave and job-satisfaction adjusting for background characteristics and facility level factors. These analyses aim to clearly specify a link between supervision methods and outcome measures with adjustments for potentially confounding factors. We contrast each supervision method (e.g. negative feedback) with formal supervision. The predictive model is common across the three models and adjusts for demographic and occupational characteristics at Level 1 and facility level intercept at Level 2, as shown in Model 1 below. Standard nomenclature is used where *i* represents the healthcare worker, and *j* represents the facility.

To identify if facility level characteristics influence the relationship between supervision methods and the two dependent variables of interest (i.e. job satisfaction and intentions to leave) we estimate a series of multilevel random intercepts and random slopes models. This set of analyses firstly involves identifying if the slope or relationship between supervision and the key outcomes varies between facilities (see Model 2 below). For example, in the case of job satisfaction Model 2 captures the extent that the facility-level slope of the relationship between the presence of formal supervision and job satisfaction varies (*u*_4*j*_) from the overall average slope in this relation across all facilities (γ_40_).

If significant variation in slopes between facilities is identified (e.g. the link between formal supervision and job satisfaction is substantially stronger in some facilities than in others) our aim is to then estimate the degree to which facility level factors may explain the variance component between the facilities. The key terms which are added to Model 1 and Model 2 to specify the random slopes and their determinants are detailed in Model 3.

Initial model:

Level 1: Job satisfaction/Intentions to leave*_ij_* = *β*_0*j*_+*e_ij_*

Level 2: *β*_0*j*_ = γ_00_+*u*_0*j*_

Job satisfaction/Intentions to leave*_ij_* = γ_00_+*u*_0*j*_+*e_ij_*

Specification of the level 1 and level 2 random intercept model:

Level 1: Job satisfaction/Intentions to leave*_ij_* = *β*_0*j*_+*β*_1_×Age*_ij_*+*β*_2_×Gender*_ij_*+*β*_3_×Occupation*_ij_*+*β*_4_×Supervision methods*_ij_*+*e_ij_*

Through substitution:

Level 1: Job satisfaction/Intentions to leave*_ij_* = γ_00_+γ_10_×Age*_ij_*+γ_20_×Gender*_ij_*+γ_30_×Occupation*_ij_*+γ_40_×Supervision methods *_ij_*+*u*_0*j*_+*e_ij_*

Adding level 2 predictors where *β*_0*j*_ = γ_00_+γ_01_W*_j_*+*u*_0*j*_

Through substitution:

Model 1: Job satisfaction/Intentions to leave*_ij_* = γ_00_+γ_10_×Age*_ij_*+γ_20_×Gender*_ij_*+γ_30_×Occupation*_ij_*+γ_40_×Supervision methods*_ij_*+γ_01_×Number of beds*_j_*+γ_02_×Facility resources*_j_*+γ_03_×Distance to referral hospital*_j_*+*u*_0*j*_+*e_ij_*

Addition of random slope to Model 1 and explaining variability in the random slope:

Model 2: *β*_4_×Supervision methods*_j_* = γ_40_×Supervisory methods*_j_*+*u*_4*j*_

Adding level 2 predictors of the link between supervision methods and job satisfaction:

Model 3: *β*_4_×Supervisory methods*_j_* = γ_40_×Supervisory methods*_j_*+γ_41_(Supervision methods * Number of beds)*_j_*+γ_42_(Supervision methods * Facility resources)*_j_*+γ_43_(Supervision methods * Distance to referral hospital)*_j_+u*_4*j*_

## Results

### Participants

In total 2,043 participants returned the survey. The largest sample was drawn from Tanzania (N = 825), followed by Malawi (N = 631), and Mozambique (N = 587). However due to missing data for some sections of the survey only 1,561 participants could be included in the analyses for the present study. The largest cause of missing data was participant's failure to indicate the supervision method employed in their facility. In Tanzania 231 (28%) participants provided no response to this item indicating that participants' could not or were not willing to classify the primary method of supervision that was in use in their facility. Fewer participants failed to indicate the supervision method experienced in their facility in Malawi (54 participants; 8.6% of the sample) and in Mozambique (40 participants; 6.8% of the sample). An additional 157 participants (7.7% of the sample) were not included in the analyses due to missing data on one or more of the remaining study variables. An error in data collection in some regions in Mozambique made it impossible to match individual to facility-level data. For this reason the multilevel models employed accounted for clustering in the data at the provincial rather than the facility level.

The average age of participants from Malawi was 34.3 (SD = 10.5), as shown in Table 1. Of this group 64.8% were female. The majority of the Malawi sample consisted of enrolled nurses (63.7%), as shown in Table 1. In Tanzania the mean age of participants was 39.76 (SD = 9.12) and 75% were female. Medical attendants were the largest group in the Tanzania sample (39%) followed closely by enrolled nurses (36%) and registered nurses (23.8%). In Mozambique participants were aged 32.1 (SD = 7.9) on average and 81.5% of those who responded to the supervision section of the survey were female. The category of nurses (61.5%) made up the bulk of the sample, as outlined in Table 1.

**Table 1:** Descriptive statistics for the demographic characteristics and adequacy of supervision, job satisfaction and intention to leave ratings from Malawi, Tanzania, and Mozambique.

Variable	Malawi	Tanzania	Mozambique
	N = 540	N = 541	N = 480
	M/N SD/(%)	M/N SD/(%)	M/N SD/(%)
Age	34.3 (10.5)	39.76 (9.12)	32.07 (7.86)
Female	350 (64.8%)	406 (75%)	391 (81.5%)
Supervision Method			
Formal	188 (34.8%)	287 (53%)	309 (64.4%)
Available on request	38 (7%)	26 (4.8%)	16 (3.3%)
Negative feedback only	116 (21.5%)	112 (20.7%)	86 (17.9%)
No Supervision	155 (28.7%)	116 (21.4%)	46 (9.6%)
Other	20 (3.7%)		23 (4.8%)
Job Satisfaction	17.22 (3.52)	16.79 (3.74)	18.17 (3.24)
Intention to leave	8.62 (2.94)	8.52 (2.6)	7.8 (2.49)
Cadre			
Enrolled Nurses	344 (63.7%)	195 (36%)	
Registered Nurses	47 (8.7%)	129 (23.8%)	295 (61.5%)
Medical attendants/Medical assistants* & Clinical officers	140 (25.9%)	211 (39%)	85 (17.7%)
Doctors	9 (1.7%)	6 (1.1%)	
Nurse Midwives			86 (17.9%)
Other			14 (2.9%)

* In Mozambique this cadre includes those who classified themselves as ‘Agent Medicine’, ‘Medical Technician’, & ‘Surgical Technician’.

### Descriptive analyses

Formal supervision with regular prescheduled meetings was the most frequently endorsed method of supervision across all three countries. Of those who responded to the supervision section of the survey 34.8% received formal supervision in Malawi, as did 53% in Tanzania and 64.4% in Mozambique (Table 1). In the absence of formal supervision participants were likely to indicate that they experience mainly negative feedback or no supervision at all. A large portion of healthcare workers in Malawi received no supervision (28.7%) with fewer in Tanzania (21.4%) and Mozambique (9.6%) (Table 1). Participants across the three countries had broadly similar levels of job satisfaction (Table 1). Almost a quarter (22.8%) of respondents in Malawi indicated that they were actively seeking other employment, whereas in Tanzania and Mozambique the percentages were 15.2% and 7.7% respectively (Table 2).

**Table 2:** Percentage of health workers in each country who have considered leaving their current position.

Intention to leave	% per country		
	Malawi	Tanzania	Mozambique
Would consider working for another Hospital/clinic	45.1%	55.8%	48.1%
Have seriously thought about leaving this hospital/clinic	33.3%	30.1%	30.1%
Am actively seeking other employment	22.8%	15.2%	7.7%

There exists greater similarity between the countries in terms of whether health workers had seriously thought about leaving their current jobs with a third of providers in Malawi and 30.1% of the sample in Tanzania and Mozambique either agreeing or strongly agreeing that they had done so. On average intentions to leave were strongest in Malawi (M = 8.62, SD = 2.94) followed closely by Tanzania (M = 8.52, SE = 2.6) with healthcare providers in Mozambique demonstrating weaker intentions to leave their current position (M = 7.8, SD = 2.49).

### Multilevel analysis

#### Supervision method, job satisfaction and intention to leave

**Figure 1:**
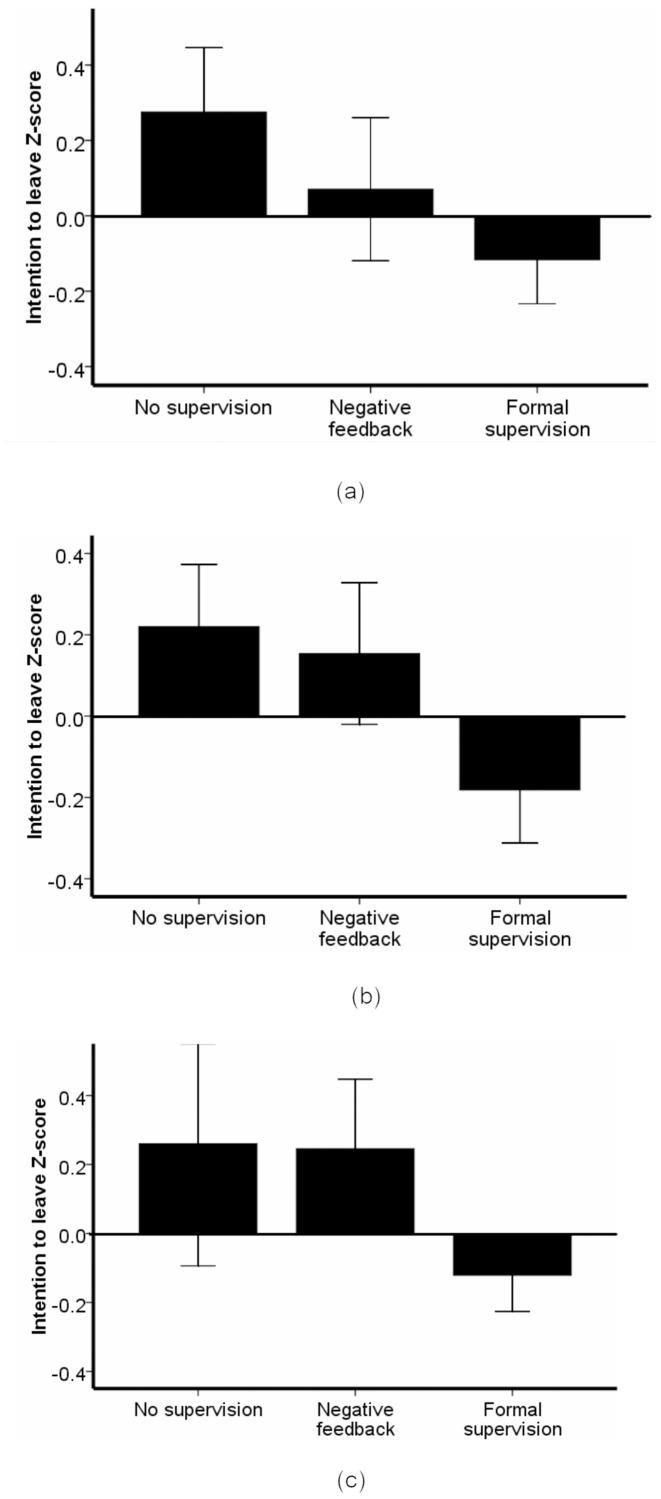
Intention to leave the workplace Z-scores as a function of an absence of supervision, negative feedback only, and formal supervision in: (a) Malawi, (b) Tanzania, and (c) Mozambique.

Not receiving any supervision appeared to be most strongly linked to decrements in intentions to leave and job satisfaction. In particular, intentions to leave were substantially increased when no supervision system was in place in Malawi (b = 1.09, SE = .31, t = 3.52, p<.01), Tanzania (b = .82, SE = .3, t = 2.73, p<.01), and Mozambique (b = 1.04, SE = .39, t = 2.67, p<.01) (Table 3 and Figure 1). There was also clear evidence of a link between the absence of supervision and diminished job satisfaction in Malawi (b = −1.07, SE = .38, t = −2.84, p<.01) and Tanzania (b = −1.66, SE = .43, t = −3.82, p<.01). Job satisfaction was not found to be related to supervision methods in Mozambique (Table 3).

**Table 3:** Results of multilevel model testing the relationship between supervision methods and job satisfaction and intentions to leave across three countries.

Supervision methods by country	Job Satisfaction		Intentions to Leave	
	b (SE)	t	b (SE)	t
Malawi				
On request	−.44 (.6)	−.74	−32 (.51)	−.63
Negative only	−1.24 (.41)	−3.04^**^	1.09 (.34)	3.22^**^
No supervision	−1.07 (.38)	−2.84^**^	1.09 (.31)	3087
Other	.25 (.79)	.32	−.32 (.67)	−.48
Base: Formal^a^				
Tanzania				
On request	−.87 (.77)	−1.12	.05 (.54)	.1
Negative only	−.74 (.42)	−1.75†	.5 (.29)	1.7†
No supervision	−1.66 (.43)	−3.82^**^	.82 (.3)	2.73^**^
Base: Formal^a^				
Mozambique				
On request	−.86 (.82)	−1.04	.94 (.61)	1.54
Negative only	−.77 (.39)	−1.97^*^	.85 (.29)	2.93^**^
No supervision	−.42 (.52)	−.8	1.04 (.39)	2.67^**^
Other	−.33 (.69)	−.49	−.47 (.51)	−.91
Base: Formal^a^				

† <.1, ^*^ p<.05, ^**^ p<.01

a Each category of supervision methods (i.e. on request, negative feedback only, no feedback) is contrasted with formal supervision in predicting the outcome variables.

Note: All analyses adjust for participant age, gender, and occupational cadre. In addition, the Malawi and Tanzania analyses adjust for facility level factors (geographic location, size, availability of resources).

Supervision characterized by negative feedback was also closely linked to negative outcomes in the three countries. Those who received only negative feedback had stronger intentions to leave their job than those receiving formal supervision in Malawi (b = 1.09, SE = .34, t = 3.22, p<.01), Mozambique (b = .85, SE = .29, t = 2.93, p<.01), and to a lesser extent in Tanzania (b = .5, SE = .29, t = 1.7, p<.1) (Table 3 and Figure 1). Supervision characterized by negative feedback predicted reduced levels of job satisfaction in Malawi (*b* = −1.24, SE = .41, t = −3.04, p<.01) and to a lesser extent in Tanzania (b = −.74, SE = .42, −1.75, p<.1) and Mozambique (b = −.77, SE = .39, t = − 1.97, p<.05) (Table 3).

As the variation between facilities in the slopes of interest was non-significant, this meant that there was no variation that could be explained through the addition of facility level moderating variables. Therefore, we rejected the hypothesis proposing that facility level variables may explain variability in the random slope of the relationship between supervision methods and job satisfaction or intention to leave the workplace.

## Discussion

In this study we utilized multilevel analyses to show that inappropriate or absent workplace supervision is a strong predictor of healthcare workers intentions to leave their position. This was the case in Malawi, Tanzania, and Mozambique The research focused on mid-level cadres who provide the majority of emergency obstetric services within the three countries under study in the belief that they are a valuable human resource that should be managed and developed in the same way as more established health cadres. Successful task shifting from established to mid-level cadres and the retention of healthcare staff in sub-Saharan Africa is contingent on the existence of adequate supervision systems [10]. Although mid-level cadres are less prone to international migration than established cadres with internationally recognized qualifications [9], anecdotal evidence suggests that they do migrate from the public to the private sector. Our study shows that about one-third of these health workers have seriously thought about leaving their current position. It appears that the absence of a supervision system may impact on staff retention amongst these cadres. The absence of supervision also appears to contribute to low levels of job satisfaction.

Supervision that consisted mainly of negative feedback was viewed to be almost as de-motivating as no supervision. Those who indicated they only receive negative supervision were more likely to state intention to leave their jobs in Malawi and Mozambique and to a lesser extent in Tanzania. Negative feedback was also linked to lower job satisfaction in both Malawi and Tanzania. These findings are consistent with literature in the area of learned helplessness theory which suggests that irregular punishment (negative supervision) from an external controlling agent is largely de-motivating [20].

Qualitative interviews with the healthcare workers reported elsewhere [21] suggest that the ‘negative feedback only’ supervision system endorsed by the participants are likely to reflect external supervision visits by the District Health Management Team. Such inspection methods are often viewed as fault finding exercises derived from a coercive ‘inspection and blame’ model of supervision. Thus, when supervision is absent or typified by negative feedback only, it is reasonable to expect that this may erode staff motivation, lead to a poor work environment and diminished performance.

The results reported detailing the relationships between no supervision or negative feedback and staff motivation reflect statistical contrasts between these types of supervision and the base category of ‘formal supervision’. Thus, we can say that those supported by formal supervision were more satisfied in their jobs and less likely to want to leave their position. These findings suggest that in resource-constrained countries supporting healthcare workers through regular pre-arranged formal supervision meetings may enhance staff motivation and retention. Taken together, our analyses examining negative supervision suggest that it is not only the presence of a supervision system that is required; rather, it must be a supervision system that ensures regular, supportive supervision available to all staff [15], [16].

One limitation of the study is that it is unclear what specific aspects of formal supervision may improve staff motivation. Cumming's systematic review suggests that people-focused leadership is an important contributor to improved motivation and performance [14]. Yet, we do not know if it is the recognition of one's performance, the process of shared problem solving, the sense of shared responsibility, or a myriad of other potential factors, that may underlie the identified benefits of regular supervision. At present, our results are therefore more informative in detailing what has failed to work rather than what contributes to a good model of supervision. Future studies could document the extent to which different forms of supervision meet the criteria for a successful supervisory process. For instance, such studies may identify if supervisors help set staff expectations regarding performance, if supervisors regularly monitor and assess staff, and if supervisors are successful in identifying problems when they occur and in realizing opportunities to take action to solve such problems [22]. A further methodological issue relating to supervision ratings is the possibility of reverse causation. In the current design, it is possible that dissatisfied staff who want to move to another position may rate the supervision they experience as absent or solely negative. Tracking participants longitudinally would assist in evaluating this possibility.

Although our results are derived from a cross-sectional survey we attempted to rigorously control for alternative explanations for the relations observed. The findings described reflect analyses that adjust for demographic and occupational factors as well as facility characteristics. We utilized multilevel analysis to show that the substantive conclusions derived from this cross-national study are robust to dependencies in data sampled from within each healthcare facility and to characteristics which differed between facilities.

## Conclusions

Improving and expanding healthcare services in resource-poor settings requires more attention be paid to the provision of formal supportive to healthcare workers. This requires positive leadership and an enhancement of the systems in place for planning, developing and supporting the workforce. This cross-sectional three country study using multi-level analysis showed that when healthcare workers are not supported their motivation suffers. Specifically, we found that a lack of supervision, or a supervision system characterized solely by negative feedback, was closely linked to intentions to leave and low job satisfaction. In contrast, when workers were supported by regular pre-arranged formal supervision meetings, they were less likely to want to leave the workplace and were more satisfied with their positions. These findings were largely consistent across health systems in Malawi, Tanzania, and Mozambique. The pervasive nature of the effects of supervision methods reflects the crucial need for more supportive human resources policies to be implemented in these three countries.

## References

[1] Gerein N, Green A, Pearson S (2006) The implications of shortages of health professionals for maternal health in Sub-Saharan Africa. Reproductive Health Matters 14: 40–50. 16713878 10.1016/S0968-8080(06)27225-2

[2] WHO (2006) The World Health Report 2006. Working together for health. Geneva: World Health Organization.

[3] Chen L, Evans T, Anand A, Ivey Boufford J, Brown H, et al (2004) Human resources for health: overcoming the crisis. Lancet 364: 1984–1990. 15567015 10.1016/S0140-6736(04)17482-5

[4] Haines A, Sanders D, Lehmann U, Rowe A. Lawn JE, et al (2007) Achieving child survival goals: potential contribution of community health workers. The Lancet, 369 (9579): 23–29. 10.1016/S0140-6736(07)60325-0 17586307

[5] Willis-Shattuck M, Bidwell P, Thomas S, Wyness L, Blaauw D, et al (2008) Motivation and retention of health workers in developing countries: a systematic review. BMC Health Serv Res 8: 247. 19055827 10.1186/1472-6963-8-247 PMC2612662

[6] Peters DH, Chakraborty S, Mahapatra P, Steinhardt L (2010) Job satisfaction and motivation of health workers in public and private sectors: cross-sectional analysis from two Indian states. Human Resources for Health 8: 27. 21108833 10.1186/1478-4491-8-27 PMC3003185

[7] Arnetz BB (2001) Psychosocial challenges facing physicians of today. Social Science and Medicine 52: 203–213. 11144776 10.1016/s0277-9536(00)00220-3

[8] Chu K, Cortier H, Maldonado F, Mashant T, Ford N, et al (2012) Cesarean Section Rates and Indications in Sub-Saharan Africa: A Multi-Country Study from Medecins sans Frontieres. PLoS One 7(9): e44484 Epub 2012 Sep 4. 22962616 10.1371/journal.pone.0044484 PMC3433452

[9] Dovlo D (2004) Using mid-level cadres as substitutes for internationally mobile health professionals in Africa: A desk review. Human Resources for Health 2 doi:10.1186/1478-4491-2-7. 10.1186/1478-4491-2-7 PMC455693 15207010

[10] Manafa O, McAuliffe E, Maseko F, Bowie C, MacLachlan M, et al (2009) Retention of health workers in Malawi: Perspectives of health workers and district management. Human Resources for Health 7 doi:10.1186/1478-4491-7-65. 10.1186/1478-4491-7-65 PMC2722569 19638222

[11] McCord C, Mbaruku G, Pereira C, Nzabuhakwa C, Bergstrom S (2009) The quality of emergency obstetrical surgery by assistant medical officers in Tanzanian district hospitals. Health Affairs 28: 876–885. 10.1377/hlthaff.28.5.w876 19661113

[12] Philips M, Zachariah R, Venis S (2008) Task shifting for antiretroviral treatment delivery in sub-Saharan Africa: not a panacea. Lancet 371: 682–684. 18295026 10.1016/S0140-6736(08)60307-4

[13] Bradley S, McAuliffe E (2009) Mid-level providers in emergency obstetric and newborn health care: factors affecting their performance and retention within the Malawian health system. Human Resources for Health 7 doi:10.1186/1478-4491-7-14. 10.1186/1478-4491-7-14 PMC2657772 19228409

[14] Cummings GC, McGregor T, Davey M, Lee H, Wong C, et al (2010) Leadership styles and outcome patterns for the nursing workforce and work environments: A Systematic Review. International Journal of Nursing Studies 47: 363–385. 19781702 10.1016/j.ijnurstu.2009.08.006

[15] Hyrkas K (2005) Clinical supervision, burnout, and job satisfaction among mental health and psychiatric nurses in Finland. Issues in Mental Health Nursing 26: 531–556. 16020067 10.1080/01612840590931975

[16] Knudson HK, Ducharme LJ, Roman P (2008) Clinical supervision, emotional exhaustion, and turnover intention: A study of substance abuse treatment counselors in the Clinical Trials Network of the National Institute on Drug Abuse. Journal of Substance Abuse Treatment 35: 387–395. 18424048 10.1016/j.jsat.2008.02.003 PMC2637454

[17] l-Dmour H, Al-Awamleh R (2002) Effects of transactional and transformational leadership styles of sales managers on job satisfaction and self-perceived performance of sales people: A study of Jordanian manufacturing public shareholding companies. Dirasat: Administrative Sciences 29: 247–261.

[18] Fernandes C, Awamleh R (2006) Impact of Organisational Justice in an expatriate work environment. Management Research News Vol 29 (11): 701–712.

[19] Bickel R (2007) Multilevel analysis for applied research. New York: Guilford Press.

[20] Martinko MJ, Gardner WL (1982) Learned helplessness: An alternative explanation for performance deficits. Academy of Management Review 7: 195–204.

[21] Bradley S, Kamwendo F, Masanja H, De Pinho H, Waxman R, et al. (under review) An in-depth exploration of health worker supervision in Malawi and Tanzania. Human Resources for Health 10.1186/1478-4491-11-43 PMC3849462 24007354

[22] Marquez L, Kean L (2002) Making supervision supportive and sustainable: new approaches to old problems. MAQ Paper No. 4 . Washington, DC: USAID.

